# Drought Stress Tolerance in Wheat and Barley: Advances in Physiology, Breeding and Genetics Research

**DOI:** 10.3390/ijms20133137

**Published:** 2019-06-27

**Authors:** Ahmed Sallam, Ahmad M. Alqudah, Mona F. A. Dawood, P. Stephen Baenziger, Andreas Börner

**Affiliations:** 1 Department of Genetics, Faculty of Agriculture, Assiut University, 71526 Assiut, Egypt; 2 Resources Genetics and Reproduction, Department Genebank, Leibniz Institute of Plant Genetics and Crop Plant Research (IPK), Corrensstr. 3, OT Gatersleben D-06466 Stadt Seeland, Germany; 3 Department of Botany & Microbiology, Faculty of Science, Assiut University, 71516 Assiut, Egypt; 4 Department of Agronomy & Horticulture, University of Nebraska-Lincoln, Lincoln, NE 68583, USA

**Keywords:** water deficit, *Triticum aestivum*, *Hordeum vulgare*, genetic improvement, selection, physiological changes

## Abstract

Climate change is a major threat to most of the agricultural crops grown in tropical and sub-tropical areas globally. Drought stress is one of the consequences of climate change that has a negative impact on crop growth and yield. In the past, many simulation models were proposed to predict climate change and drought occurrences, and it is extremely important to improve essential crops to meet the challenges of drought stress which limits crop productivity and production. Wheat and barley are among the most common and widely used crops due to their economic and social values. Many parts of the world depend on these two crops for food and feed, and both crops are vulnerable to drought stress. Improving drought stress tolerance is a very challenging task for wheat and barley researchers and more research is needed to better understand this stress. The progress made in understanding drought tolerance is due to advances in three main research areas: physiology, breeding, and genetic research. The physiology research focused on the physiological and biochemical metabolic pathways that plants use when exposed to drought stress. New wheat and barley genotypes having a high degree of drought tolerance are produced through breeding by making crosses from promising drought-tolerant genotypes and selecting among their progeny. Also, identifying genes contributing to drought tolerance is very important. Previous studies showed that drought tolerance is a polygenic trait and genetic constitution will help to dissect the gene network(s) controlling drought tolerance. This review explores the recent advances in these three research areas to improve drought tolerance in wheat and barley.

## 1. Introduction

Drought stress can be simply defined as a shortage of water which induces dramatic morphological, biochemical, physiological, and molecular changes. All of these changes reduce plant growth and crop production. wheat (*Triticum aestivum* L.) and diploid barley (*Hordeum vulgare* L.) are among the most important cereal crops and large portions of human populations in many parts of the world depend on them as a source of food and animal feed. Both crops can be grown in a wide range of agro-climatic environments, however, many of these environments have drought stress as one of the major challenges to their production and productivity. In 2013, approximately 65 million ha of wheat production was affected by drought stress [1]. The predicted global warming and climate change will increase the frequency of drought, hence the losses of the agriculture crop productivity.

Drought stress can occur at any growth stage and depends on the local environment. Therefore, genotypes may be tested for their drought tolerance at relevant and often different growth stages because some genotypes may tolerate drought at germination or seedling stage, but these may be very sensitive to drought at the flowering stage or vice versa. Drought tolerance is determined by identifying a trait that can be used to measure the effect of drought stress on plants. This trait should discriminate tolerant and susceptible genotypes. Hence, it is very important in any drought experiment to determine the appropriate trait(s) that are drought-tolerant traits. Furthermore, drought tolerance and yield should be improved in parallel because farmers need to profitably produce their agricultural products under drought stress.

In the past, many researchers have studied drought tolerance in barley and wheat, but the improvement of these crops for drought tolerance is limited for many reasons. First, drought may cause dramatic changes in the physiological parameters in the plant which need to be measured and understood. Second, genotypic × environment (GE) interaction will affect selection. Third, drought is a complex trait controlled by many genes, most of which make a minor genetic contribution, but these are important to genetically improve drought tolerance. However, other factors are also related to crop such as the structure and complexity of the wheat genome. Drought stress can be studied in different aspects through physiological, morphological, breeding, gene expression, or genetics studies. To maximize the understanding about drought tolerance, the integration of various information and methods from different research fields is recommended and research collaborations from these fields must be integrated. 

When plants are exposed to drought stress, they physiologically change to tolerate this stress. Physiologically, drought needs a context-dependent view to understand the ability of plants to make important changes that alleviate the effect of drought stress [2]. Drought-tolerant plants try to have less reduction in water content, membrane stability, and photosynthetic activity. The tolerant group tries to accumulate soluble sugars, proline content, amino acids, chlorophyll content and enzymatic and non-enzymatic antioxidant activities [3]. Plant physio-morphological traits are very important for selection in a breeding program to improve drought tolerance due to their relation to the adaption for future climate scenarios [4]. Moreover, identifying the genes controlling these physiological changes may lead to rapid genetic improvement for drought tolerance in a plant. 

Plant breeding research is very important to produce new wheat and barley cultivars having a high degree of drought tolerance. In addition, to improve drought tolerance, plant breeders must improve grain yield combined with high tolerance to drought. The first step is to select the potential germplasm that contains genotypic differences for drought tolerance [5]. Breeders choose whether they test the germplasm at a specific growth stage or multiple growth stages based on their climate and the objective of the study. The selected traits are scored on all elite genotypes to define the drought tolerance. Then, the selection is based on drought tolerance and yield. After identifying/selecting a group of tolerant genotypes, a breeding program may start by crossing the selected genotypes as donor parents. Breeders can use any trait (morphological or physiological or yield related-trait) to improve drought tolerance but there must be a few trait(s) that can discriminate between drought tolerant and drought susceptible lines, have high heritability estimates, and along with a positive significant correlation with final grain yield [6].

The recent advances in genomics make whole-genome sequencing for each genotype possible. One of the most and widely used method is genotyping-by-sequencing which generates large numbers of single nucleotide polymorphism (SNP) markers that cover the wheat and barley genomes [7,8]. Moreover, the reference genomes for barley and wheat are available for imputation. These genome references allow identifying the accurate position and the location on the chromosome for each SNP generated by genotyping-by-sequencing (GBS). This huge number of SNPs is used for genome-wide association study (GWAS) and quantitative trait loci (QTL) mapping to dissect the genetics of complex traits by identifying genomic regions or genes as possible controlling target traits, in this case, drought tolerance. The number of genes identified depends on the number of measured traits that are associated with drought tolerance and the magnitude and proximity of the genes. The more traits scored leads to the identification of many genes controlling drought tolerance [9,10]. It is very important also to identify the number of genes controlling drought tolerance in each selected genotype. There are many specific molecular markers for important drought genes such as *Dreb* and *Fehw3* genes [11]. Therefore, the presence or absence of these two genes can be tested in any germplasm. The genetic improvement for drought tolerance can be achieved by identifying new genes controlling drought using GWAS or QTL mapping [12,13]. Furthermore, genomic selection and gene editing can be used for improving drought tolerance in wheat and barley [14].

Combining information from the three research areas; physiology, breeding, and genetics may help to identify the most drought-tolerant genotypes having the highest number of genes controlling drought tolerance. This review explores the recent advances of physiology, breeding, and genetic research for improving drought tolerance and the possible ways to identify the promising drought-tolerant genotypes for further genetic improvement for this trait.

## 2. Physiological and Biochemical Responses

**Figure 1:**
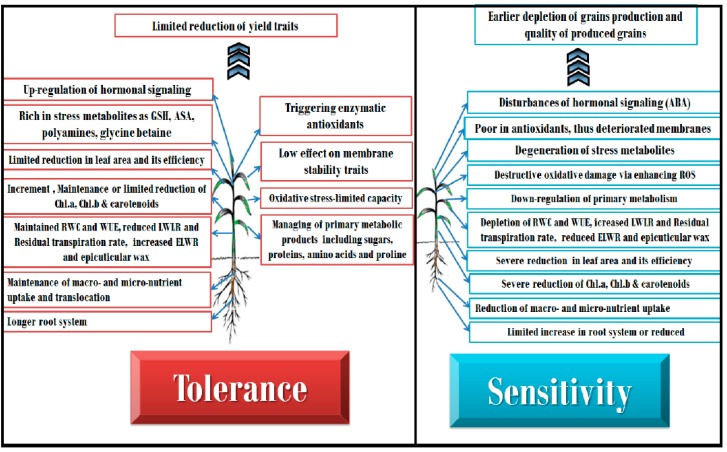
Physiological changes in tolerant and susceptible wheat and barley genotypes in response to drought stress.

In past, various physiological and biochemical responses have been identified in response to drought stress. There are many important physiological traits that alleviate the effect of drought stress on wheat and barley plants. Genes controlling these physiological changes are very important for geneticists and breeders as they are useful sources to genetically improve drought tolerance through a breeding program. The major physiological changes that occur in tolerant and susceptible wheat and barley genotypes are illustrated in Figure 1. 

### 2.1. Photosynthesis and Gaseous Exchange

Photosynthesis is the main driver of grain yield and plant growth. Hence its role in understanding the physiological basis of a plant’s response to drought is critical. Variation in photosynthetic pigment contents is the key indicator to determine the extent of photosynthesis in plants grown under water stress conditions. It is well known that drought decreases the photosynthetic rate of cereals [15]. The major components limiting photosynthetic rate are the CO_2_ diffusional limitation due to early stomatal closure as a response to the drought-induced loss of turgor, reduced activity of photosynthetic enzymes, the biochemical components related to the triose-phosphate formation and decreased the photochemical efficiency of photosystem II [16]. Metabolic distortions of photosynthetic activity could be due to an imbalance between light capture and its utilization [17], decrease in Rubisco activity, loss of chloroplast membranes [18], degradation of chloroplast structure and photosynthetic apparatus, chlorophyll photo-oxidation, destruction of chlorophyll substrate, inhibition of chlorophyll biosynthesis, and the increase of chlorophyllase activity [19]. However, the drought-induced limitations of photosynthesis through metabolic distortions are more complex than stomatal limitations which mainly occur through the reduced synthesis of photosynthetic pigments [20]. Stomatal and mesophyll conductance to CO_2_ often decrease in response to drought [21]. Stomatal closure limits transpirational water loss and aids plants to conserve water status under drought stress. Nonetheless, closure of stomata, in turn, results in decreased CO_2_ availability for photosynthetic carbon metabolism, declines net CO_2_ assimilation rate and prohibits plants ability for dry matter accumulation [22]. Drought affects photosynthesis pigments differentially depending on species or genotype studied. Genotypic variation-differentially affected chlorophyll content. Genotypes with high chlorophyll content resulted in better seed yield under water-deficit conditions [23]. Positive correlations of grain yield in wheat with chlorophyll content, grain filling period, and the number of grains per spike were reported [24]. In barley, grain yield under late drought stress was positively correlated with grain filling duration and gross photosynthetic rate [25]. Furthermore, the tolerant wheat cultivars enhanced total chlorophyll at pre- and post-anthesis stage accompanied by a more stable photosynthetic rate, while susceptible cultivars reduced both traits for both stages [15]. Chlorophyll has a crucial role in plant energy production, thus the susceptible plants suffer from insufficient energy needed for normal growth. Thus, breeders and genetic workers should select the wheat and barley cultivars which are able to sustain photosynthetic apparatus and photochemical efficiency under deficit irrigation for a limited reduction of grain yield.

### 2.2. Water Relations

Water content, relative water content, succulence index, water loss rate, excised leaf water retention and residual of transpiration rate are some important characteristics that influence plant water relations. Relative water content (RWC) is a measure of plant water status, reflecting the metabolic activity in tissues and used as the most meaningful index for dehydration tolerance. A decrease in the RWC in response to drought stress has been noted in a wide variety of plants [26]. Grain yield of barley was negatively correlated with leaf water potential under drought stress conditions [25]

The change in water loss in terms of excised leaf water loss may estimate the plant’s leaf water relations, especially when comparing fully hydrated leaves to those under deficit irrigation and it is presumably an indirect measure of cuticular thickness and cuticular transpiration [27]. This trait greatly reflects the balance between water supply to the leaf and transpiration rate. The genotypes with reduced the excised leaf water loss are believed to be more drought tolerant, less affected by evapotranspiration water losses, therefore able to conserve their water content [28]. Drought stress enhanced excised leaf water retention (ELWR) which reflect the water retention mechanism in the leaf under stress that may be ascribed to leaf rolling or decrease in exposed leaf surface area, hence the increase in ELWR could be a superior indirect selection criterion for drought tolerance leading to higher grain yield [29]. A significant positive correlation was found between relative water content and grain yield under drought stress during the reproductive stages in wheat and barley. Therefore, RWC and leaf rolling could be used for selection in breeding programs to improve drought tolerance in a combination with high yielding [30,31]

To control the water loss associated with epidermal conductance, plants developed epicuticular waxes which are the organic compounds of the cuticle which covers the outer surface of plant tissues. It was found by [32] that epicuticular wax might be an important attribute in drought tolerant genotypes because they developed more epicuticular wax on leaves which reduced the loss of water from the plant leaf surface. Reduction of residual transpiration rate associated with the drought tolerance in crop plants and has been used as a selection criterion in wheat and barley breeding programs [33]. Agronomic parameters like photosynthetic rate, RWC, and stomatal conductance show strong positive correlations with water use efficiency, whereas transpiration rate expresses negative correlation with WUE under drought [34]. Leaf waxiness and trichome density may lessen water loss and protect against drought for longer periods. Moreover, crops or genotypes ascertained low ELWL, low residual transpiration rate, and high ELWR under drought have a higher capacity to preserve water balance in their leaves reflects their drought stress tolerance, thereby higher yield stabilization.

### 2.3. Nutrient Relations

Drought-induced reductions in uptake and translocation of macro-nutrients (N, P, and K^+^) have been reported in various plant species [35] presumably due to reduced root volume and in dry soils, the nutrients are not available. Water limitations accompanied by low N is the main constraint to wheat yield which affected the leaf–water relations, chlorophyll fluorescence and photosynthetic processes leading to restricted plant growth rate, early senescence, reduced grain filling duration with limited grain weight and poor crop productivity [36]. As the water content in the soil decreases, the radius of water-filled pores decrease, tortuosity increases and P mobility decreases [37]. A decline in available P reduces P uptake and consequently reduces foliar P content [38]. Moreover, drought stress reduces the active transport and membrane permeability of cations (K^+^, Ca^2+^, and Mg^2+^), thus resulting in decreased absorption of these cations via roots [39]. Drought stress tended to decrease Ca^2+^ concentrations in the aboveground biomass and this effect was attributed to the reduction in transpiration flux [40]. A similar reduction of the levels of calcium, potassium, and phosphorus in roots and shoots of the wheat plant under water stress was recorded by [35]. Drought can induce the deficiencies of some micro-nutrients, i.e., Mn, Fe, and Mo [41]. However, these micronutrients become increasingly available under well-watered conditions due to their conversion to more soluble and reduced forms for uptake [42]. Thus, deficit irrigation modified plant ionic homeostasis via decreasing their availability, uptake, and translocation besides deactivating the metabolic pathways of nutrients in plants. The symptoms of nutrients deficit were co-responses to drought stress especially chlorosis. Also, maintenance of macro-and micro-nutrients is of promising criteria for tolerant cultivars under deficit irrigation which should be taken into consideration by breeders. 

### 2.4. Oxidative Status

#### 2.4.1. Reactive Oxygen Species (ROS)

Reactive oxygen species (ROS) can be singlet oxygen (^1^O_2_), superoxide radicals (O_2_), hydrogen peroxide (H_2_O_2_), and hydroxyl radical (OH) resulted in oxidative damages to plants. The presence of ROS causes alteration of the cellular redox potential which gives rise to oxidation of photosynthetic pigments, membrane lipids, proteins, and nucleic acids, thereby triggering cell death, lessening plant growth and productivity [43]. However, the adverse effects of drought stress are based on its duration, timing, and magnitude of stress [43]. ROS production is linear with the severity of water stress that triggered the peroxidation of membranes, organelles and enzyme activation or inactivation and breakdown of nucleic acids [44]. The increase in the content of malonic dialdehyde (MDA) has been considered as a suitable marker for membrane deteriorations. A previous study reported that the decrease in membrane stability reflects the extent of lipid peroxidation caused by ROS [45]. Low MDA levels were associated with drought stress tolerance in wheat [46]. It is worth mentioning that increased lipoxygenase enzyme activity (LOX) is responsible for the oxidation of polyunsaturated fatty acids and thus enhances lipid peroxidation under stress conditions [47]. There is a differential accumulation of LOX activities under drought stress, compared to non-stressed plants [48]. A similar relationship between increased LOX activity and oxidative stress were also observed [47]. Enhanced lipid peroxidation and ROS compromise cell membrane functions resulting in loss of membranes’ ability to control the rate of ion movement in and out of cells which often are used as a test of damage to a great range of tissues. More leakage for metabolites or ions means a greater damaged membrane which was caused by sensitivity to drought. The increment of electrolyte leakage measurements was evaluated as an evaluation test for cell damage degree for nine wheat genotypes [44]. The cell damage index revealed an important genotypic difference which may help to discriminate between genotypes showing similar responses regarding to other physiological and/or biochemical parameters. Reactive nitrogen species (RNS) are slightly diverse than ROS. The increase in the uncontrolled production of ROS and RNS may provoke modifications in macromolecules that can act as markers for both oxidative stress (lipid peroxidation and protein carbonylation) and nitrosative stress (lipid nitration, protein tyrosine nitration, and S-nitrosylation). Superoxide radical and nitric oxide are used to generate peroxyinitrite, a powerful oxidant that can mediate the tyrosine nitration of proteins which might be an effective biomarker of nitrosative stress in higher plants [49] 

Another stress metabolite induced in response to drought stress, methylglyoxal, which accumulates in plant cells during normal physiological processes like photosynthesis; however, its levels dramatically elevated under various abiotic stresses [50]. It is toxic to plant cells, causing inhibition of cell proliferation, degradation of proteins and inactivation of antioxidant defense systems and consequently disrupts cellular functions [51]. A higher amount of methylglyoxal production under drought and salinity stresses was reported [52,53,54]. 

#### 2.4.2. Antioxidant System

Production of antioxidant enzymes such as catalase (CAT), superoxide dismutase (SOD), peroxidase (POD), ascorbate peroxidase (APX), monodehydroascorbate reductase (MDHAR), dehydroascorbate reductase (DHAR) and glutathione reductase and glutathione peroxidase (GPX) in response to water stress has been shown to be a well-known adaptive mechanism in wheat and barley. In barley, the expression pattern APX, CAT and SOD depend on the plant development stage and genotype under drought stress [55]. Under drought stress, a significant increase in the expression pattern of genes encoding CAT, APX, and GPX enzymes was observed in drought-tolerant wheat genotypes. These genes could play a very important role in controlling drought stress in the wheat genome [56]. The tolerance of some genotypes to environmental stresses has been associated with higher activities of antioxidant enzymes as illustrated in Table 1. For instance, the drought-tolerant species of wheat had higher activities of SOD, POD, and CAT than the drought-sensitive species [57]. Wheat plants subjected to mild drought enhance leaves’ APX activity, whereas prolonged water deficit decreased its activity due to the increased production of MDA [58]. Tolerant wheat genotypes had a high POD activity, high phenolic contents and a low damage index indicating greater stomatal closure [44]. The activities of the detoxification-related enzyme GST enhanced in wild barley under water-deficit irrigation [59]. Glutathione reductase (GR) enzyme plays an important role by maintaining reduced glutathione (GSH), ascorbate (AsA) pools and properly reduced glutathione (GSH)/oxidized glutathione (GSSG) ratio that is more decisive in determining plant resistance to abiotic and biotic stresses than in the actual GSH content [60]. The elevated level of GR reduces the rate of electron flow to O_2_ inducing the formation of O_2_^•−^ and the metal-catalyzed formation of ^•^OH, through Haber-Weiss reaction [61]. Genetically engineered plants overexpressing *MDHAR* and *DHAR* genes had greater protection against abiotic oxidative stress and a higher level of AsA content in the leaf tissues and other plant organs [60].

Deficit irrigation induced an increment in total and reduced ascorbate contents of two barley cultivars [65]. Moreover, the accumulation of phenolic compounds against abiotic stresses including drought has been described in wheat [44]. A significant increase was recorded in flavonoids and phenols in flag leaves of wheat plants under deficit irrigation which might be owing to the antioxidant role of flavonoids and phenolics which minimized potentials and accessibility of ROS under drought-induced oxidative stress and improving plant protection by a lipid peroxidation reduction. Anthocyanin, as a water-soluble pigment belongs to the family of phenolic compounds, are usually rather resistant to drought [67] that is related to superoxide radical scavenging activity and of anthocyanins ability to stabilize the water potential. Callose plays important roles in a variety of processes in plant development and in response to multiple biotic and abiotic stresses. An earlier study demonstrated that water shortage increased callose content in all wild barley genotypes because drought protection is mainly induced by abscisic acid, which might be coupled with callose deposition [59]. As callose could hinder the plants’ defense machinery against drought and/or salinity by increasing its water-holding capacity, it might also have a higher water use efficiency in the Tibetan wild barley genotype during the vegetative stage. Furthermore, Chitinases are other components of plant defenses, and their expression is induced in plants by environmental and biological stresses. The expression of the *Chi2* gene was increased as confirmed by chitinase activity which may help ameliorate drought and salinity tolerance in Tibetan wild barley [68]. Most ubiquitous polyamines (PAs) in plants are putrescine (Put), spermidine (Spd) and spermine (Spm) are small positively charged molecules, which are involved in the response to drought [69]. They stabilize membranes, regulate osmotic and ionic homeostasis, and act as antioxidants and interact with other signal molecules. Under drought stress conditions, higher PAs contents in plants are related to increased photosynthetic capacity, reduced water loss, improved osmotic adjustment and detoxification. PAs accumulation is the immediate response observed after exposure to drought conditions in barley [70]. Furthermore, carotenoids are necessary for photo-protection of photosynthesis and they play an important role as a precursor in signaling during the plant development under abiotic/biotic stress. Growth improvement in plants under stressful environment has been widely reported to be due to the significant role of zeaxanthin in alleviating oxidative damage of membranes [71]. Water stress has been shown to affect a number of other phytochemicals, including α-tocopherol which is a lipid-soluble antioxidant associated with the biological membrane of cells, especially the membrane of the photosynthetic apparatus. α-tocopherol has been reported to be involved in the suppression of peroxidation of membrane lipids by reducing the MDA content and thus protecting the integrity of the bio-membranes [72]. Accordingly, the activity of one or more antioxidant enzymes generally increases in plants exposed to drought could work coordinately or synergistically to prevent cellular damage, and this elevated activity correlates with increased drought tolerance. 

The identification of genes that encode such enzymatic activities under drought stress in wheat and barley is very important in a breeding program that aims to use and study many wheat and barley genotypes. All the above studies use two or few genotypes to identify the expression of genes. Specific primers can be designed for these genes and can be used in screening hundreds or thousands of genotypes in a breeding program to improve drought tolerance in barley and wheat using marker-assisted selection. 

### 2.5. Osmotic Balance 

Adaptation of plants to water-deficit classified into three categories: drought escape, dehydration avoidance, and dehydration tolerance or its combination. Osmolyte accumulation is one of the drought tolerance mechanisms which allows cells to manage their dehydration and membrane structural integrity to give tolerance against drought and cellular dehydration [73]. Osmotic adjustment in plants exposed to drought may follow storage of low-molecular-weight organic solutes. The wheat plant accumulates several inorganic and organic solutes in its cytosol to lessen its osmotic potential for the maintenance of cell turgor [73]. Under drought stress, plants produce and accumulate compatible solutes such as sugars, polyols, and amino acids to facilitate osmotic balance and water absorption and retention [74]. Carbohydrates play multiple functions on osmoprotection, osmotic adjustment, carbon storage, detoxification of reactive oxygen species, protection of membrane integrity, caused the protection of macromolecules and DNA structures and stabilization of enzymes/proteins. In extreme dehydrated states, sugars become an essential replacement for water, even more than proline, providing a hydration shell around proteins [4]. Wheat genotypes accumulate more soluble sugars during the grain filling period than the pre-anthesis stage under drought stress [75]. On the other hand, the reduction of total soluble sugars could be ascribed to water induced loss of solutes (mainly K^+^) from guard cells, which resulted in a selective reduction in guard cells turgor leading to stomatal closure [76].

Proteins are compounds of fundamental importance for all functions in the cell [77]. In this regard, the declared impaired protein synthesis accompanied with a reduction in the plant growth and the crop yield under water stress condition which is due to the reduced number of polysomal complexes in tissues with lower water content [19]. In addition, the generation of ROS caused the oxidation of amino acids and could burst the protein structure under drought stress. However, a significant relationship was observed among total proteins and grain yield of wheat under rain-fed conditions [75]. On the other hand, an increase in shoot proteins of the wheat plants cultivated under water stress condition was observed [35]. The drought stress-induced proteins allow plants to make biochemical and structural adjustments that enable plants to cope with the stress [78]. 

The presence of proline is one of the common traits in most of the cereals under drought [79]. Wheat plants accumulate proline than the other osmoregulators, especially in leaves as a consequence of the increasing collapse of proteins with an immediate decline in its synthesis during the grain filling stage under water deficit [80]. It is osmotically active, controls storage of useful N, and plays a major part in membrane stability. It also helps by scavenging free radicals and buffering cellular redox potential which helps wheat plants to combat abiotic stresses. As a signaling controller molecule, it initiates many mechanisms that help in adaptation to drought [81]. However, few plant species can produce enough proline to greatly reduce the abiotic stress effects [82]. 

Drought stress also alters the endogenous levels of glycine betaine which shields cells from water deficit by preserving the osmotic balance between extra and intracellular environments, increasing the quaternary structure of proteins, e.g., antioxidant enzyme protection and membrane proteins and the oxygen releasing complex of photosystem II [83]. It also regulates intracellular osmotic potential, controls the pH of cytoplasm, and stabilizes cell membrane structure of wheat in drought stress [84]. 

The changes in osmotic balance differ from genotypes. For example, the ability of proline accumulation in response to drought depends on the genotype. The genetic variation of such osmatic changes could be very useful in improving drought tolerance in wheat and barley in selection programs (e.g., selecting the genotypes having a higher proline content under drought stress than under normal conditions) [85].

### 2.6. Hormonal Effect

Abscisic acid production can affect drought adaptation through both dehydration avoidance and dehydration tolerance (Thompson et al., [86]). Abscisic acid (ABA) is the most critical hormone involved in regulating tolerance to abiotic stresses such as drought, salinity, cold, heat and wounding [87]. ABA has long been acknowledged as a major chemical root-to-shoot stress signal [88], inducing inhibition of leaf expansion and short-term responses like stomatal closure. ABA is involved in the regulation of systemic responses to abiotic stress before there are any detectable changes in leaf water or nutrient status [89]. Moreover, ABA was found in wheat to act as a promotor for root growth which has a significant correlation with yield under drought stress [90]. Osmotic stress results in the synthesis or catabolism of several other growth regulators, including auxin, cytokinins, ethylene, gibberellins, brassinosteroids, jasmonic acid and other factors (e.g., nitrogen, pH) that have been shown to be involved in the regulation of physiological processes through their action as signal molecules in signaling networks [91]. ABA controls plant growth by refining root development and modifying leaf elongation and expansion during water deficit [92]. Abscisic acid regulates tissue water content through stomatal oscillations and induces the expression of genes encoding proteins that control cellular dehydration tolerance [93]. Previous reports suggest that under drought ABA synthesis occurs in xylem tissues, which is then transported to reproductive organs where it may influence grain filling by modulating the expression of genes involved in carbohydrate metabolism and cell division. Accumulation of ABA in leaves and stem or root exudates, upon exposure to drought, increases with simultaneous reductions in leaf cytokinin contents [94]. Reduced ethylene and 1-aminocyclopropane-1-carboxylic acid concentrations and increased ABA concentration in developing wheat grains under mild drought increased the grain-filling rate. However, under severe drought, ethylene, ACC, and ABA concentrations were too high, reducing the grain-filling rate [95]. Moreover, gibberellin A3 (GA3) application to the roots restored leaf elongation in semi-dwarf and tall genotypes growing in restrictive soil; the longest leaves were attained when GA3 was applied to affected roots of tall genotypes [96]. In this sense, the plants up-regulate endogenous hormones to withstand the harsh conditions especially cytokinins and ABA and related hormones to hasten the deleterious impacts of water stress on plants. 

Assessing hormone accumulation, response and hormonal ratio provide an effective tool for selecting the promising drought tolerant wheat genotypes. Different genotypes may present different sensitivity to drought via hormone responses. A set of six spring wheat lines was phenotyped for ABA and ethylene. High genetic variation was found among genotypes for ABA and ethylene which have an association with yield [97]. Therefore, genes controlling hormones accumulation under water deficit can be used for improving drought tolerance in wheat and barley. 

As described above, there are a lot of physiological changes when plants are exposed to drought stress. These changes may include an increase or a decrease of the physiological components. The ability of tolerant plants to response for drought tolerance depends on the genotype. Therefore, the genetic variation in these physiological changes should be studied to select the true drought-tolerant genotypes. Such genetic variation in the physiological traits is very useful in breeding wheat and barley for improving drought tolerance.

## 3. Advances in Breeding for Drought Tolerance

Drought tolerance is a very complicated trait and one that can be approached from different aspects. Breeding drought tolerant lines requires useful assays to select for drought tolerance, a key aspect of successful plant breeding. The assays may be stage-specific (e.g., for emergence, for grain number, or during the grain filling period if that is when drought generally occurs). Initially, drought-tolerant wheat and barley genotypes can be selected based on drought-tolerance traits. The selected genotypes are crossed to try to incorporate multiple resistance genes for drought stress, which will be selected for enhanced drought tolerance. Traditionally, breeders depend on phenotypic selection for the trait of interest. For drought tolerance that could be drought tolerance per se (direct selection) or a related trait(s) (indirect selection) that is more heritable or easier to identify. Generally, breeding for improved drought tolerance in cereals must be combined with good yield potential [98] because there are occasional seasons with above-average moisture. From these crosses, plant breeders select elite progeny for drought tolerance. Selection for drought tolerance must be tested in more than one year or/and location in the target environments because the drought tolerance usually has low heritability. Furthermore, drought tolerance measurements are often affected by spatial variation, so the trials need multiple replications. The efficiency of phenotypic selection is also affected by GE interaction if the environments are different (which is expected due to year to year or site to site variation). High levels of G × E can lead to no progress for drought tolerance because the environments require selecting for different types of drought tolerance. Therefore, the G × E interaction is considered a major complication in breeding programs. To overcome the low heritability of drought tolerance, plant breeders have integrated DNA molecular markers into their programs with good impact in improving drought tolerance in cereals [12]. These breeders have: (1) detected genomic regions controlling drought tolerance through quantitative trait loci (QTL) mapping, and (2) revealed the genetic diversity among the elite genotypes at that region by marker polymorphism. Drought tolerance is a polygenic trait controlled by many genes. Most of these genes have minor effects [99]. The QTL mapping has detected many genomic regions, with minor and major effects, associated with drought tolerance in cereals. Testing these particular genomic regions, after validation, associated with drought tolerance can be used for improving drought tolerance by screening hundreds or thousands of genotypes for the presence or absence of these genomic regions. While expensive, this result will save a lot of time and effort. Identifying the target genomic regions using QTL mapping or GWAS depends on the number and type of DNA markers and the phenotypic assay. The more DNA markers, the more coverage of the genome, and the higher the probability to detect as many QTLs as possible for drought tolerance. For the type of DNA marker, simple sequence repeats (SSR) are co-dominant markers and widely used in QTL mapping. Recently, advances in DNA sequencing has provided new techniques for genotyping by producing high-density single nucleotide polymorphism (SNP) markers [100]. Genotyping-by-sequencing is becoming one of the most important sequencing methods and it can provide a hundred thousand SNPs that can cover the whole genome of wheat and barley. Combining traditional breeding programs with the advances of DNA sequencing made great progress in targeting the important genes controlling drought tolerance in wheat and barley [7,13,101,102].

### 3.1. Genetic Variation of Drought Tolerance at Different Growth Stages

Climate change will have a great impact on increasing the effects of drought stress in the agricultural sector by limiting the production and productivity of the important agricultural crops (e.g., wheat, barley, etc.). Drought stress can occur in any growth stage and without irrigation. The severity of drought stress entirely depends on the environment in which the drought occurs. Therefore, it is very important to have previous meteorological data on the occurrences of drought before designing a breeding program to improve drought tolerance. The growth stage at which genotypes are tested for drought tolerance should be carefully considered. For example, improving seedling traits that are associated with drought tolerance may not be appropriate if the drought stress occurs during seedling growth and development stages, but it will be useless if drought stress only occurs around the flowering or grain filling stage [103]. Also, breeding of drought tolerance is often affected by other factors in dry environments (e.g., erratic weather patterns, soil-borne diseases, soil mineral nutrition, etc.). Genetic variation in drought tolerance can be studied under controlled conditions in greenhouses or growth chamber and under field conditions. The main issue in any field experiment with drought stress is affected by many other factors also in dry environments, including erratic weather, heat stress, soil mineral nutrition, etc. Therefore, testing drought tolerance under controlled conditions is useful but mainly to augment working in the field where many factors are not controlled [103]. As climate changes affect the weather and, hence, the ability to predict the weather in a particular environment will be decreased. As a result, drought may occur in a grain filling stage instead of occurring at the seedling stage due to the effect of climate in a respective environment. Accordingly, it is preferable and recommendable to evaluate the same genotypes under controlled and field experiments to select the promising genotypes for target traits [104]. The traits that are used to define drought tolerance differ by growth stage. The current study focused on the most important growth and development stages in wheat and barley, namely; germination, seedling, reproduction, and grain filling to understand and study the genetic variation in drought tolerance. 

#### 3.1.1. Drought Tolerance at the Germination Stage

Seed germination is a series of events that starts with water imbibition and it ends when the radical emerges from the seed coat [105]. It is a sensitive stage to drought stress which can reduce germination and seedling emergence in wheat and barley. For example, in India and Pakistan, rainfed dry areas are planted with an expectation of the coming monsoon season [106]. Therefore, if rain does not occur after sowing, the germination will be negatively affected. Dissecting the natural variation and genetic base of germination and related traits under drought stress will improve barley and wheat growth and yield. Germination experiments usually are conducted in the lab using the controlled conditions. For example, to study the effect of drought stress at germination, breeders might want to simulate high osmotic stress. This assay can be done by creating an elevated osmotic potential which is needed to study the genetic variation to the elite genotypes in drought tolerance. A solution of polyethylene glycol (PEG) can be used to induce drought stress that is measured using a timescale of days after treating the seeds with the PEG solution. There are many different concentrations of PEG, therefore, it is essential to test a wide range of concentrations [107]. PEG has a high molecular weight (6000 or 8000) and prevents water from penetrating the cell wall. Hence, the PEG is used to control water potential in germination experiments [108]. Basically, in germination experiments using PEG, the seeds of genotypes are tested to different concentration (e.g., 5, 10, 20 %, etc.). Breeders focused on the ideal concentration at which they can distinguish among the tested genotypes for their drought tolerance. The most tolerant genotypes that can germinate at this concentration are recommended to be re-evaluated on higher concentrations as a further test to select among the most drought-tolerant genotypes. The basic traits for measuring the germination under drought tolerance are germination rate, germination percentage (G%) and germination pace (GP). Previous studies also estimated both traits under normal conditions in order to estimate the reduction in G% and PG due to drought stress [12]. However, there are few related traits that can be scored in a germination test such as shoot length, root length, and shoot: root ration of germinated seeds [8]. 

#### 3.1.2. Drought Tolerance at the Seedling Stage

**Figure 2:**
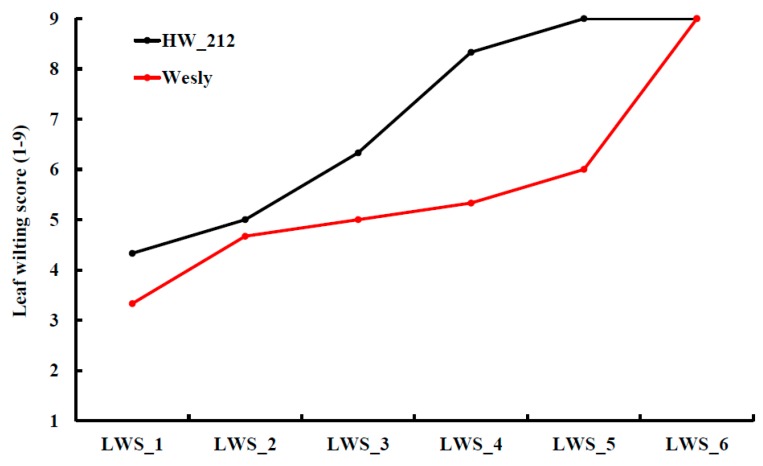
Phenotypic variation between HW—212 and Wesley in leaf wilting symptoms during drought treatment. Anoton is the tolerant check (American cultivar) [8].

**Figure 3:**
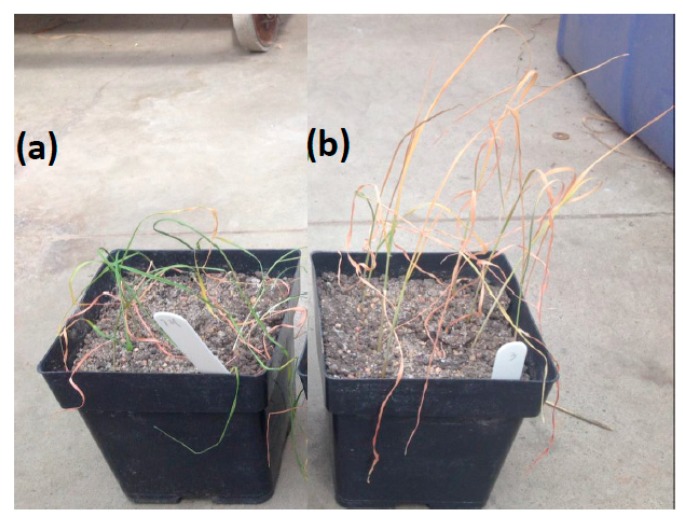
Difference between two genotypes in their leaf wilting and stay green traits (**a**) a genotype had green leaves and was fully wilted; and (**b**) a genotype had yellow leaves and no leaf wilting.

**Figure 4:**
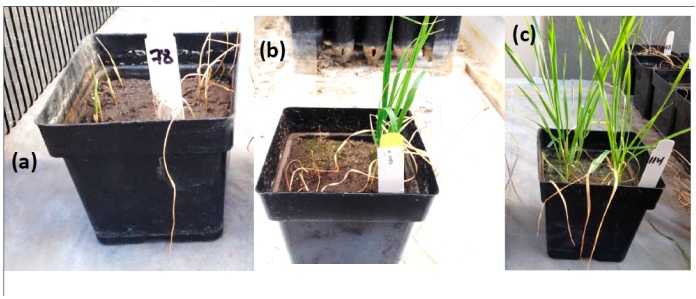
Responses of genotypes to drought stress. (**a**) a genotype had a little regrowth after rewatering and the leaves if un-cutting plants were not recovered; (**b**) a genotype did regrew after rewaterd but the leaves of un-cutting plants were fully recovered; (**c**) a genotype had a good regrowth and leaf recovery after rewatering.

Seedling stage, the next stage after germination, is when a plant develops more than traditional cannabis leaves. It is also a very critical stage to moisture stress. In many regions with low precipitation after the optimum sowing date, it is a critical stage for drought stress, and when drought stress often occurs. Evaluating drought tolerance at the seedling stage is very important because it affects all the subsequent stages and ultimately grain yield [109]. By studying genetic variation at this stage, it is possible to increase the selection intensity in breeding for drought-tolerant varieties [110]. During this stage, genetic variation studies in drought tolerance focused mainly on the leaf and root characteristics. Understanding the correlation among these traits is very important to improve the efficiency of breeding for drought tolerance in wheat and barley. The most important question here is how few of the many possible traits can be used to define and select the most drought tolerant genotypes. The most common definition of drought tolerance is the ability of the plant to tolerate prolonged water deficit and endure low relative leaf water content [111]. This definition can be studied by scoring leaf wilting, days to wilting, and stay green traits. These traits can be visually scored and are considered morphological traits. However, these traits can be also good indicators of physiological changes. Leaf wilting and days to wilting refer to the loss of leaf water content, while stay green refers to the loss of chlorophyll content. A new protocol for breeding drought tolerance at the seedling stage was tested on a bi-parental population [103]. The authors scored eight traits and divided them into two categories based on the definition of drought, (1) tolerance traits which address the ability of plants to endure prolonged water deficit, and (2) recovery traits that address the ability of plants to recover and regrow after prolonged water deficit and re-watering. In this protocol, leaf wilting (LW) was visually scored (1 = no wilting, 9 = fully wilted) six times after water withholding until the end of drought, then all scores were summed up to form a trait named as sum of leaf wilting (SLW) which reflected the effect of drought stress on plant leaves during drought treatment [103]. Many earlier studies scored leaf wilting one time at the drought treatment. However, scoring subsequent leaf wilting during drought treatment has an advantage of evaluating drought tolerance of the respective genotype over time precisely. For example, Wesley and HW_212 genotypes were scored nine for LW at the end of drought treatment (sixth score) Figure 2. HW_212 started to be fully wilted at the fifth scoring date with a wilting degree of 8.5 on average (after 17 days from water withholding), while, Wesley was scored as 6 on average on that date. Although both genotypes were scored as susceptible at the end of drought treatment, Wesley was less affected by drought stress than HW_212 based on SLW (33 versus 36 for Wesley and HW_212, respectively) [103]. Therefore, Wesley could have a better recovery if it is re-watered. SLW is more informative than LW. Days to wiling (DTW), scored as the number of days from water withholding until the first wilt of leaves was also an important trait that reflected the time in which the genotypes started to respond to water deficit. Stay green was a good indicator for drought tolerance, and was scored in many drought experiments in wheat [4,112,113,114]. Stay green and leaf wilting are controlled by different genes and there was no correlation among these two traits [115] (Figure 3). Leaf fresh matter, leaf dry matter, and relative water content are basics traits that are widely used in drought experiments in wheat and barley. In the second group of traits (recovery traits), four recovery traits namely; days to regrowth, regrowth biomass, leaf recovery after drought, and drought survival trait were scored by [103]. Half plants of each genotype were cut after exposing to 21 days of drought stress to score the ability of plants to regrow after re-watering (simulating the end of the drought period). The other half was kept measuring the recovery of plants after a drought. Days to regrowth as a trait was estimated as the number of days from cutting until each cutting plant started to produce the first leaf. These traits provided information on the different response of the genotypes to re-watering after prolonged drought stress. For example, cut plants of some genotypes had little regrowth after re-watering, but the uncut plants for the same genotypes did not recover (Figure 4a). For other genotypes, cut plants did not regrow after re-watering, but the uncut plants recovered well (Figure 4b). An example of a good genotype that regrew and recovered after re-watering after prolonged drought stress is illustrated in Figure 4c. Although, the traits in each group (tolerance and recovery groups) were well phenotypically and genotypically correlated, there was no promising correlation between the two groups. The lack of correlation between recovery and tolerance trait is that the traits are genetically independent, and breeders must select for both groups of traits (e.g., selecting for drought tolerance does not select for recovery and vice versa). To overcome this problem, a selection index [116] was created for each group, namely: tolerance index which included SLW and DTW and recovery index which included regrowth biomass, days to regrowth, and drought survival rate. The correlation between the two indices was also non-significant. Moreover, the tolerance index did not have any correlation with individual recovery traits and no correlation was found between the recovery index and individual tolerance traits. The two indices were combined to form the drought tolerance index (DTI) which had a highly significant correlation with all tolerance traits, recovery traits, tolerance index, and recovery index. The most promising drought tolerant genotypes, for both types of traits, were then identified and selected. The advantages of creating the DTI was to combine both information obtained from both groups of traits so that selections could be made efficiently and with high significant phenotypic and genotypic correlations and high heritability estimates. 

Previously, tolerance and recovery traits had a highly significant correlation under frost stress in faba bean (*Vicia faba* L.) [117]. The relationship will differ by stress tolerance. In drought experiments at the seedling stage, it is recommended to (1) consider both groups of trait and (2) test the elite genotypes for as many traits as possible in each group to select the most promising drought tolerant genotypes [103]. From the genotypic and phenotypic correlations, it can be determined if both groups of traits are controlled by different genes. As the protocol was tested in a bi-parental population, a preliminary QTL mapping experiment revealed two different major QTLs were detected. Each QTL controlled a different group of traits [118].

As the seedling stage is very sensitive to drought, wheat and barley breeders should select the traits that address the main aspects of drought tolerance at this stage.

#### 3.1.3. Drought Tolerance at Flowering and Grain Filling Stages

Drought stress also occurs during flowering and may extend up to grain filling which affects the number of seeds per spike and kernel weight, two important components of grain yield. As a grain yield is a complex trait controlled by many genes, breeders often use indirect selection and use well-correlated traits with the yield for improving grain yield in dry environments [119]. Yield traits that breeders have used for assessing drought stress on wheat or barley plants include seedling vigor, plant height, days to heading, days to maturity, spike length, number of spikelets per spike, root architectural traits, number of grains per spike, thousand kernel weight, grain yield per spike, grain yield, biological yield, and harvest index. Drought tolerance as a trait can be assessed from any of these traits or from drought indices which accurately assess the genotypic yield response to drought stress [120]. There are two common ways for evaluating drought tolerance by sowing the elite genotypes under normal and dry environments; (1) estimate of the reduction in a trait due to stress for each genotype using the following equation:

(1) $$Reduction\;in\;a\;triat\;due\;to\;drought\;stress=\;\frac{Xn-Xd}{Xd}\;\times \;100$$

where *X_n_* and *X_d_* is the main performance of the genotype under normal and dry environment for a particular trait, respectively.

(2) Drought susceptibility index (DSI) for each genotype which can be used as follows 

Firstly, drought intensity (DI) was estimated according to [121] as follows:

(2) $$DI=1-\frac{Y_{d}}{Yn}$$

where *Y_d_* is the average all genotypes for the respected trait (under drought stress), while, the *Yn* is the average of all genotypes for the same respected trait under well-watered conditions. The drought susceptibility index (DSI) is estimated for each genotype according to [121] as follow

(3) $$DSI=\frac{1-\frac{X_{d}}{X_{n}}}{DI}$$

where *X_d_* is the mean performance of each genotype for the respected trait under drought environment, while, the *X_n_* is the mean performance of each genotype for the same respected trait under well-watered conditions. 

**Figure 5:**
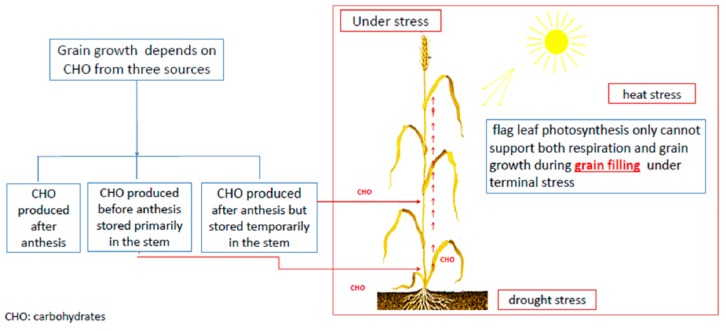
The three souses of carbohydrates that are transferred into grains during grain filling under drought and heat stresses.

Moreover, there are some important traits which can be scored in this stage and have a strong relationship with drought tolerance such, as flag leaf persistence, leaf rolling, canopy temperature, and stomatal conductance. These traits refer to the ability to reduce evaporation loss and maintain photoassimilate production [122]. Stem characters play an important role in grain weight under terminal stresses such as drought and heat. Stem density, stem weight, and stem diameter were measured and positively correlated with grain yield per spike (GYPS) and thousand-kernel weight (TKW) [123]. High-positive significant phenotypic and genotypic correlations were found between TKW and stem diameter (r = 0.56 **), and stem weight (r = 0.39 *). Also, GYPS was correlated with stem diameter (0.54 **), stem density (r = 0.61 **), and stem weight (r = 0.44 *) [119,123]. To understand the relationship between stem characters and grain weight under drought stress, it is important to know the sources of carbohydrates that support grain growth and development in wheat (Figure 5). Three main sources that the carbohydrates availability can be obtained from (I) post-anthesis synthesis and directly transferred to the grains, (II) post-anthesis synthesis, but stored temporarily in the stem before remobilization to the grains, and (III) pre-anthesis synthesis stored primarily in the stem and remobilized to the grains during the grain-filling stage [124]. When wheat and barley plants are exposed to drought or heat stresses during grain filling, photosynthesis rapidly declines which reduces the available assimilates to the grain. Consequently, a dramatic reduction in kernel dry weight occurred [125]. Furthermore, the wheat canopy respires quickly during the grain filling stage adding more demand on photosynthesis [126]. As a result, flag leaf photosynthesis alone cannot support grain growth and respiration under drought or heat stresses [4]. Therefore, a considerable amount of stored carbohydrates in wheat is needed during grain filling and must come from reserves assimilated pre-anthesis [127]. Hence, stem traits in wheat and barley such as stem length, stem weight, and internode specific weight can affect accumulation and mobilization of stem reserves with maximum specific weight appear to be correlated with stem-mobilized dry matter [128]. Moreover, the amount of remobilization was found to be in a linear relationship with single stem dry weight at anthesis under drought stress [129]. 

Breeders can test the genotypes in the same environment, in which irrigation can be controlled, for their performance under well-watered and drought stress [119,123]. Many environments rely on rainfall, therefore, breeders often select low rainfall environments with irrigation to test genotypes under drought stress and compare the same set of genotypes in well-watered environments [103]. Moreover, a breeding program for improving drought tolerance differs from environment to environment based on the performance of genotypes and is measured by GE. 

### 3.2. High-Throughput Phenotyping for Improving Drought Tolerance in Wheat 

High-throughput phenotyping (HTP) is a new technology that can be used for rapidly screening thousands of genotypes for many traits. This technology needs a highly automated facility in greenhouses or growth chambers with good environmental controls, accurate sensing techniques, and robotics [130] or phenocarts or unmanned aerial vehicles (UAVs, syn. drones) in the field. The ability to screen thousands of genotypes for a particular trait can accelerate plant breeding process because it generates a previously unavailable and useful data simultaneously in a detailed and non-invasive manner for traits that related to drought stress such as leaf temperature, plant water status, and predicted yield level [131]. These platforms include sensor systems (passive or active spectral sensors) which allow the estimation of various vegetation indices and plant parameters [132,133]. HTP is also designed to measure plants grown in the field. Phenotyping of the genotypes for drought tolerance under field conditions is very challenging due to the association between the decline of soil moisture and the increase of mechanical impedance [130]. Another issue is that the plants in the field are normally exposed to other stresses also, hence there may be confounding of different applied stresses. Therefore, it is difficult to mimic the field environment under controlled conditions. Even using HTP in the field, phenotyping remains a major issue limiting the advances in a breeding program to improve drought tolerance. In addition, the choice between phenotyping under open field conditions and controlled greenhouse will depend on the objective of phenotyping and the heritability estimates of the traits [134]. High-throughput phenotyping was used to evaluate genotypes for traits that are associated with drought tolerance in wheat and barley such as seedling vigor, seminal root traits, and physiological traits [135,136,137].

The main hindrance to using this technology is the cost and skilled labor which many institutes cannot afford. Most breeders only can score the basic traits to evaluate drought tolerance under field or controlled conditions. Often these traits such as leaf rolling, stay green, leaf wilting, etc. are visually scored as an inexpensive attempt to incorporate physiological assays into plant breeding. 

### 3.3. The Use of Nanotechnology in Improving and Breeding Drought Tolerance

Recently, plant breeders have become interested in agricultural nanotechnology, which can be defined as the application of nanoparticles (NP) which may have some beneficial effects to the crops, with its tools to enhance productivity and tolerance to various biotic and abiotic stresses tolerance [138]. Three advantages of using nanotechnology are: they are cheap, of low consumption, and of low phytotoxicity [139] though nanoparticles may have positive and negative biological effects based on their concentration [140]. Nanoparticles created by green synthesis, which is considered a natural repository of green elements in the form of animal-derived biomaterials, phytochemicals, and biomolecules of microbial or plant origin, have less toxic effects compared to those produced by chemical or physical synthesis [141]. The use of an appropriate concentration of NP can increase the adaption of plants in stressful conditions [139]. For example in barley, drought tolerance was studied using the application of SiO_2_ and TiO_2_ nanoparticles during reproductive stages under field conditions [142]. The application of SiO_2_ improved yield components under drought stress, while TiO_2_ decreased the seed yield components at some concentrations [142]. 

Different concentrations of titanium dioxide nanoparticles (TiO_2_-NPs - 0, 0.025, 0.05, 0.1, 0.2, and 0.5%) were used to identify the concentration which stimulated the seeds germination percentage and other seedling traits of four wheat cultivars [143]. They found an increase in root length, shoots length, chlorophyll content and other seedling traits at the concentration of 0.1%, while, no improvement was found at a concentration of 0.5%. Sakha93 genotype had the highest response to TiO_2_-NPs. The effect of zinc and copper nanoparticles on pro-oxidative balance, the content of photosynthetic pigments, leaf area was studied in two wheat varieties (Stolichna and Acvedic) at seedling stage to improve drought resistance [139]. The results revealed a significant increase of antioxidative enzyme activity which reduced the accumulation of thiobarbituric acid reactive substances. The nanoparticle also stabilized the level of photosynthetic pigments during drought stress and increased leaf water content, increasing drought tolerance in wheat. Notably, both genotypes responded to nanoparticles differently under drought stress which can be explained by a genetic variation which could be used for breeding to improve drought tolerance in wheat. 

However, there are many considerations that should be taken into account before breeders incorporate NPs such as the concentration and type of NPs and how they relate to their use in agriculture. It is important to understand the effect of nanoparticles on the environment and on the genes and the interaction between NPs and genotypes. The effect of nanoparticles on important yield traits should also be studied to know whether the nanoparticles, that induce stress tolerance, have a negative impact on some important traits such as flowering time, grain quality, and grain weight. It is essential to understand the genetic changes in the response to nanoparticles. Wheat root tips were exposed to different silver nanoparticle (AgNPs) concentration (10, 20 40, and 50 ppm) [144]. The AgNPs interfered with the cell’s normal function and caused chromosomal aberrations such as incorrect orientation at metaphase, chromosomal breakage, metaphasic plate distortion, spindle dysfunction, stickiness, aberrant movement at metaphase, fragmentation, scattering, unequal separation, scattering, chromosomal gaps, multipolar anaphase, erosion, and distributed and lagging chromosomes. 

From the viewpoint of breeding research, the different response of genotypes to the safe concentrations of nanoparticles can be used as a source of genetic variation. The effect of this nanoparticle should be extensively studied on other important yield traits. Dissecting the molecular genetics changes is quite needed to understand the action of these nanoparticles. 

As the main task of breeding research is to explore the genetic variation in which can be used to improve drought tolerance, it is very important to identify genes controlling such genetic variation to genetically improve drought tolerance in wheat and barley.

## 4. Genetic Landscape of Drought Tolerance in Wheat and Barley

Understanding the genetics behind drought stress tolerance as a quantitative trait influenced by genetic with many quantitative trait loci (QTLs) and environmental factors are remains a challenge for plant biologists and geneticists [145]. Drought tolerance is a complex trait as it is usually accompanied by heat or other abiotic stresses that lead to different morphological and physiological changes [146,147]. Adaptation processes to drought stress conditions involve the genetics of these confounding factors at the molecular, physiological, biochemical and biological levels and processes [147]. Genetic control of drought tolerance traits related requires intensive and integrative genetic, genomic and molecular researches to determine the genes underlying them and in which stage and mechanism or process they are involved. Elucidation of the genetic and molecular mechanisms underlying drought tolerance in wheat and barley will ultimately lead to developing drought-tolerant varieties [8].

### 4.1. The Genetic Basis of Drought Tolerance 

Genetic analyses of drought tolerance have been studied through the development of molecular markers and genome sequencing in wheat and barley. Such analyses include several approaches e.g., QTL-mapping, association-mapping, genome-wide analyses, and expression analysis aim to identify QTL or gene-related traits to adaptation drought stress [146]. Revealing the genetic basis underlying the drought tolerance in wheat and barley requires a phenotypic and genetic variation of relevant traits in large populations with dense genetic maps. The complexity of the genetic basis of drought tolerance is due to polygenic inheritance, the small effect of QTL, and high GE, hence low-heritability. Furthermore, the genetic independence of drought tolerance at different developmental stages makes the detected QTL less useful in crop improvement. Therefore, several QTLs have been discovered for drought tolerance related traits, but a limited number of QTLs are genetically characterized or cloned and incorporated in breeding programs [145]. Utilizing genetic analyses, approximately 800 QTLs for drought-tolerant traits (agronomic, physiological, root, and yield-related traits) have been identified in wheat, of which ~700 and ~110 QTLs and MTAs were detected by bi-parental-mapping and GWAS respectively [148]. The number was less in barley with ~ 500 QTLs [149] and ~90 [8], respectively. Finding large-effect, stable QTL that controls many drought tolerance related traits at different developmental stages would be a great effort for crop improvement, but has not been found. Currently, the advances of wheat and barley genome sequencing with the state-of-the-art bioinformatics tools are helping QTL mapping and linking the minor effect QTLs into the physical position on the genome that has led to candidate gene prediction and characterization.

#### 4.1.1. Quantitative Trait Locus (QTL) of Drought Tolerance

Dozens of important genomic regions have been detected using the classic QTL-mapping approach. This research helped identify the loci underlying the variation of drought tolerance related traits and elucidating the genetic factor of this complex trait in wheat and barley. Multi-environmental field conditions are commonly used to evaluate the genotype performance [150,151] using a different type of bi-parental population e.g., recombinant inbred line (RIL) population [150,151,152,153], doubled haploid (DH) population [154,155] or advanced backcross [156]. Different DNA molecular markers (restriction fragment length polymorphisms (RFLPs), amplified fragment length polymorphisms (AFLPs), simple sequence repeats (SSR) [152,154] and single nucleotide polymorphisms (SNPs) [156]) have been used to genotype the populations and identify QTL. Recently, a high-density genetic SNP map (from and SNP array or genotyping by sequencing (GBS)) have been used to genotype the population [155]. To understand the genetic basis of drought tolerance required strong statistical models that include the phenotypic and genotypic variation. Initially, simple interval mapping (SIM) followed by composite-interval mapping (CIM) [14] and multi-environment QTL mixed with regression models [157] have been effectively used.

The influence of drought stress on plant performance, development and yield can be determined by dissecting traits across the plant life cycle. Such analysis helps to define the QTL of the most sensitive trait and/or stage to drought stress, and whether there are shared drought tolerance QTLs among the developmental stages with traits and with final yield (Table 1). Despite this, drought stress has an impact on seed germination, vigor and seedling development [158], few studies identified the genetic basis of drought tolerance at early vegetative developmental stages using QTL-mapping population in wheat [159] and barley [160]. Also, there has been a little success in identifying the genetic basis of drought stress during the highly sensitive reproductive phase for determining the final grain [161]. Most of the QTL studies have focused on the final grain yield components (Table 1) under drought stress conditions in wheat and barley [154]. For example, many QTLs have been detected for grain yield on chromosomes one, three and six [162,163,164], grain number per spike on chromosome two, three and six [162,165,166] and spikelet number per spike on two, five and six [167]. Such major QTL controlling grain yield can be used in marker-assisted selection breeding for yield improvement under drought stress. While these MTAs are important for breeders it is also important to understand if these MTAs also relate to drought tolerance at the reproductive stage which is tightly associated with final grain yield [168]. QTL studies using a bi-parental mapping population have also discovered the genetic factors of other physiological and adaptive traits (Table 2) e.g., leaf chlorophyll content, leaf waxiness and leaf rolling in barley [155], transpiration efficiency, water-use efficiency, biomass, leaf area, and growth rate related traits [169] transpiration efficiency in wheat [170]. Interestingly, QTLs on chromosome two, four and five for leaf rolling and leaf chlorophyll content are syntenic between wheat and barley (Table 2). Meta-QTL (MQTL) analysis on drought tolerance in wheat has revealed QTLs for, photosynthesis, soluble carbohydrates, water status, carbon isotope discrimination, canopy temperature, coleoptile vigor and stay-green [149].

The plant accumulates ABA under drought stress [29], and QTL of such trait can help in understanding the drought-tolerance mechanism. Seven QTLs were identified for ABA content in wheat under drought stress of which the 5A QTL had the largest effect [181]. This QTL was coincident with the QTLs that also encodes for drought tolerance as predicted by carbon isotope ratio, chlorophyll content and flag leaf rolling [172]. Proline content is another metabolite that is considered in a drought tolerance mechanism and four QTLs have been detected in barley F_2_ population under drought stress whereas the strongest QTL was located on 5H [179]. Finally, many QTLs have been detected using barley DH and RIL populations for root related traits under drought stress conditions [180] which were also validated by MQTL [183]. Even though dozens of potential QTLs have been identified in wheat and barley for drought tolerance-related traits, very few have been validated or utilized in breeding programs for improving yield under drought stress. For a rare example, the desired alleles from some QTLs for several drought-related traits have been incorporated in breeding programs for the improvement of drought tolerance in Indian wheat elite cultivars [184]. The common QTLs between wheat and barley are promising in marker-assisted selection (MAS) since their effectiveness has been tested in different locations, in different drought conditions, and in different genera.

#### 4.1.2. Genomics Analyses of Drought Tolerance 

Recently, genome-wide analyses include genome-wide association study (GWAS) and genomic selection (GS) has been used to understand the genetic complexity of and breed for drought tolerance. GWAS approaches can be used with large numbers of SNPs that produce a high-dense map in a large and diverse collection that provides an alternative approach to identify specific genes whereas the GS can be used in both bi-parental and diverse populations.

GWAS demonstrated its strength to detect novel loci and genes for drought tolerance in wheat and barley. For example, GWAS revealed QTLs for yield component traits in 208 genotypes of durum wheat using 6, 211 SNPs [13], in 93 bread wheat genotypes using 16,383 DArTs [102], and in 123 wheat cultivars using the 90K SNP array [185]. Many significant genomic regions for grain yield-related traits have been detected using these diverse collections and marker types (Table 3) e.g., 2B, 3A and 3B [36,37,38]. The genetic architecture and candidate genes of drought tolerance-related traits including yield, leaf, and root were predicted using 108 bread wheat with 9646 SNPs [10] and in 200 bread wheat genotypes using 20,881 SNPs [186]. These studies identified important genomic regions controlling many traits under drought stress conditions (Table 3) e.g., 1A and 6B are significant regions highly associated with grain yield, root, and leaf architecture [187], of which the 1A region had been detected for root traits by bi-parental mapping [188]. A limited number of studies have focused on physiological traits e.g., leaf green area, leaf water content and water-soluble carbohydrates with around 12 MTAs have been detected [189]. Chromosome 1A was also found to contain an important genomic region for physiological traits such as water-soluble carbohydrates [9,44,45,190]. Very recently, [191] used the latest wheat genome sequences to physically map the most consistent and important genomic regions that associated with many agronomic and physiological traits under drought stress in wheat (Table 3). For instance, the physical region of 1A (516732460- 522189599) was as a highly significant region for grain weight, flag leaf area and flag leaf width [39]. Out of the aforementioned GWAS studies, only two studies used bioinformatics analysis to predict candidate genes [186]. The predicted candidate genes were involved in agronomic and physiological drought response traits, hence provide good candidates for molecular breeding to improve drought tolerance, however, none of these genes underwent for further genetic and/or molecular characterization and validation.

In barley, although many bi-parental mapping studies have been conducted to detect MTAs of drought tolerance related traits, our knowledge of genetic understanding of drought tolerance agronomic and physiological related traits at different developmental stage/phase using GWAS is still limited. Very recently, several MTAs within the significant genomic region (QTLs) of drought tolerance during seed germination have been detected (Table 3) in 218 diverse barley accessions using 9000 gene-based SNPs [8]. Among these associated QTLs with seed germination parameters and seedling related traits, some are very close to candidate genes which are located on 1H (46–48 cM), 2H (12.7, 112–114, 118–120 cM), 5H (44–45 cM) and functionally know as drought tolerance encoding different transcription factors [8]. Remarkably, shoot and root length at early developmental phase are sharing the same genetic region i.e., 1H (46–48 cM) with seed germination [8] and proline content [198]. During the vegetative phase, many QTLs have been associated with physiological traits under drought stress especially leaf senescence at 2H (49.2 cM), 5H (44.2 cM), 7H (128.3 cM), among them 5H containing candidate genes that are known to be involved in leaf senescence [196]. GWAS was also conducted during the reproductive phase to study the genetic basis of agronomic and physiological traits under drought stress (Table 3) using 148 European barley [50] and 107 six-rowed diverse collections [198]. Interestingly, 3H (125–127 cM) and 6H (95–96 cM) genomic regions were reported to be highly associated with flag leaf length and grain number and spike length respectively (Table 3) [193]. Barley studies found important drought tolerance QTLs (Table 3) which are highly associated with the shoot, root length during germination, spike length, flag leaf sheath length and peduncle length at 2H (10–14 cM), germination percentage, internode length, flag leaf length 2H (118–122) [8,193]. Moreover, allelic variation at the genomic region of 5H (44–50 cM) controlled many drought tolerance related traits e.g., germination and its reduction [6], biomass [48], water use efficiency, water content and relative water content [197]. In barley, the yield components under drought using a GWAS approach have received less attention (Table 3) because of the difficulties in phenotyping large populations and lack of efficient experimental designs. For instance, very few studies used GWAS to investigate the genetic basis of yield components in a diverse barley collection under drought stress using DArT, SSR and SNP markers [192]. Integrating genetic and physical maps of SNPs and other markers will lead to a high-density map and the ability to use all of the available information in molecular breeding for drought tolerance.

Genomic selection (GS) has just emerged in wheat and barley as one of the important approaches for predicting genotype performance and that applied to breed for drought tolerance. The few studies using this approach to obtain genomic estimated breeding values (GEBVs) found that they were between 0.4–0.50 for grain yield indicating the contribution of synthetic wheat genotypes in improving grain yield under drought stress [200]. Estimating the GEBVs for the drought tolerance related traits will be a valuable resource for the genetic improvement and yield-boosting under drought stress conditions.

#### 4.1.3. Functional Validation of Drought-Tolerance QTLs and Candidate Genes 

Functional validation and cloning of predicted candidate genes underlying drought tolerance QTLs have encountered obstacles since most of the QTLs are not ‘stable’ in different environments, were developed using different marker types (DArT, SSR, AFLPs and SNPs) and were mapped in populations using different parents. Hence, it was often difficult to obtain a precise genetic position. In addition, the small population size and markers used in the previous QTL studies led up to wide QTL intervals. Additional difficulties arose from a large number of genes controlling drought tolerance, GE, and large genome size in wheat and barley compared with other cereals like rice. Using the recent advances in genomic and next-generation sequencing will help to align the sequence of the previous stable QTLs to one reference genome to obtain their physical positions that will make the analysis more accurate and then narrow down the QTL region to predict candidate genes and accelerate the positional-gene cloning. The application of the improved genome sequencing should ultimately lead to the identification of homologs/orthologues of drought tolerance loci/genes underlying the genetic basis of drought-tolerance traits that can be used for breeding. 

Genes encoding many transcription factor (TFs) family members have been identified as involved in drought tolerance e.g., *DREB, NAC, WRKY, MYB, bZIP, TZF* in addition to protein kinases e.g., calcium-dependent protein kinases (CDPK), mitogen-activated protein kinases (MAPK) and protein phosphatases [122,201]. Six wheat genes encoding MYB TF were cloned in wheat (*TaMYB16, TaMYB24, TaMYB31, TaMYB74, TaMYB77,* and *TaMYB78*) which are *Arabidopsis* orthologues of drought-responsive genes involved in the regulation of cuticle biosynthesis and flag leaf development [202]. Moreover, the orthologue of many calcium-dependent protein kinases (CDPKs) has been identified in barley based in silico and expression analyses which demonstrated the involvement of CDPKs in signaling pathways in response to drought [202]. The *HvP5CS* gene, encoding delta-1-pyrroline-5-carboxylate synthase (P5CS), had been cloned in barley as the main drought-tolerance gene [203]. 

Cloning will become more effective and routine work with the utilization of high-throughput and accurate phenotyping and genotyping. GWAS provides many candidates for gene-based association mapping encoding many TFs which are involved in drought tolerance and need to be validated and cloned (Table 3). For instance, 26 and 61 candidate genes were for agronomic and physiological traits, respectively under drought stress of which many genes encoded *WRKY, MYB, bZIP, MAPK*, and protein kinase that were found to be associated with leaf and root architecture related traits and grain yield (Table 3) [191]. Out of 33 candidate genes found to be associated with drought tolerance traits in barley during early developmental phases [8,199], three germination-related drought tolerance genes encoding protein phosphatases and *TZF* (Table 3) were detected [8]. Further genetic and molecular validation of these candidate genes can contribute significantly to drought tolerance.

With recent advances in the marker development era, it is possible to genotype several candidate genes using KBioscience competitive allele-specific polymerase chain reaction (KASP) assay with a polymerase chain reaction (PCR). Such an approach was successfully applied in wheat and barley to detect and validate the genes e.g., grain yield and drought-tolerance genes [12,61]. Kompetitive allele specific PCR (KASP) results demonstrated its power in QTL and gene validation for drought tolerance in diverse and mapping populations. For instance, two KASP markers were designed for two important genes controlling drought; *Dreb* and *fehw3* [11]. Therefore, KASP is recommended for high-throughput marker screening of a large number of functional genes in wheat and barley that can accelerate the characterization of parents and their progenies and diverse collection of MAS. 

#### 4.1.4. Genetic Engineering of Drought-Tolerance Genes in Wheat and Barley 

One of the main goals of genetic engineering is to produce stable inheritance and expression of drought-tolerant plants carrying single or multiple-desired traits in the following generations. For instance, water-use efficiency, biomass accumulation, and root weight were improved under drought stress in transgenic wheat lines by expressing the barley *HVA1* gene [204]. Transgenic wheat lines also were improved by having more osmoprotectant through transferring a mannitol biosynthesis (mtlD) gene from *Escherichia coli* [205]. The wheat transgenic lines showed high tolerance to salt and drought stresses by *TaERF3*-overexpression [206] and significantly higher yield by transforming TaDREB3 from ‘Bobwhite’ [207]. Overexpressing *TaDREB2* and *TaDREB3* in barley transgenic lines had increased drought tolerance through protecting cells from desiccation and damage [208]. Interestingly, overexpression of *HvSNAC1* in barley improved drought tolerance and other biotic stresses e.g., fungal infection of *Ramularia cello-cygni* [209]. Overexpression of *TaNAC2* transgenic Arabidopsis plants enhanced abiotic stress tolerance, including drought. Therefore, transgenic plants have the potential for use in breeding to improve abiotic stress tolerance. Application of new genome editing technologies such as Clustered Regularly Interspaced Short Palindromic Repeats associated protein 9 (CRISPR-Cas9) in improving drought tolerance had been demonstrated in maize under field conditions [195]. Using CRISPR Cas9-based genome editing with high-quality wheat and barley reference genomes should certainly improve drought tolerance and yield. 

## 5. The Path Forward: Identifying the Most Drought-Tolerant Genotypes for Further Improvement of Drought Tolerance

Identifying and selecting the true drought-tolerant genotypes is a challenge. As mentioned previously genotypes respond differently to drought tolerance at different growth stages, however, the need is for stage independent drought tolerant genotypes. The key point of identifying the most drought-tolerant genotypes is the phenotyping as that is where the producers must see the benefit. The following steps are suggested to identify target genotypes. 

First, plant material selected for evaluating drought tolerance plays an important role in the identification of useful parents and genes. Plant material could be a diverse population or bi-parental population from carefully selected parents. For the bi-parental population, the parents may present a contrast in drought tolerance (tolerant vs. susceptible) or different mechanisms of drought tolerance. A good example of that first population is ‘Harry’ (drought-tolerant) and ‘Wesley’ (drought-susceptible) winter wheat bi-parental population [210]. Parents of the bi-parental population could be drought tolerant genotypes, but they should be genetically dissimilar (low genetic similarity) to expect segregation for drought-tolerance genes. For example, two frost-tolerant parents were crossed to form a bi-parental population. The frost tolerance was segregated in their F_10_ RILs and many QTLs for frost tolerance were detected [211]. 

Second, plant materials should be phenotyped accurately using an appropriate assay and trait that has a direct relation to drought tolerance. Single-trait evaluation for drought tolerance to distinguish between tolerant and susceptible genotypes is not recommendable. Instead, breeders and physiologists should score as multiple traits if possible. Each trait will provide useful information on drought tolerance such as tolerance and recovery traits scored at seedling stage in wheat. Each growth stage has specific traits that can be measured. Most of these traits are morphological traits. Physiological traits should be included with any traits scored at any growth stage [212]. Then, breeders perform selection based on the most tolerant common genotypes for each trait scored in their study. For example, 11 traits associated with drought tolerance at the seedling stage in wheat [103]. The researchers selected the best 20 drought tolerant genotypes for each trait. The common genotypes were selected. The results revealed one genotype that was among the best 20 genotypes in nine traits. A selection index can be calculated to include more than the target trait as described in [123].

Third, after phenotypic selection of the most drought-tolerant genotypes, the next step is for geneticists and molecular breeders to test the association between DNA markers and all traits (morphological, yield, physiological traits, etc.) scored in their plant materials. The association, that may be detected using GWAS or QTL mapping based on the population of the study, will identify new possible genes and explain epistasis. It is also highly recommended to genotype the same plant material for well-characterized genes controlling drought (e.g., possibly *Dreb*, *fehw3*, validated QTL, etc.) to test the presence or absence of major drought tolerance genes in tested genotypes. Geneticists and molecular breeders should investigate the genome of each genotype, that was phenotypically selected for drought tolerance, to identify how many genes and QTL that each genotype possesses. Then, selection should be for those genotypes that include the highest number of genes and QTL controlling drought tolerance. 

Fourth, parents for future crosses can be selected based upon the complementation of the drought-tolerance genes identified above to continue the pyramiding or stacking of drought-tolerant genes. The crosses and progeny will have higher numbers of genes controlling drought tolerance.

## Figures and Tables

**Table 1:** List of important enzymatic activities that are increased and activated in wheat and barley under drought stress.

Traits	Crop	Reference
Superoxide dismutase	Wheat	[62]
	Barley	[63]
Glutathione reductase	Wheat	[64] Shan et al. 2018
Glutathione peroxidase	Wheat	[64,65]
Ascorbate peroxidase	Wheat	Shan et al. 2018
	Barley	[63]
Monodehydroascorbate reductase (MDHAR)	Wheat	Shan et al. 2018
Dehydroascorbate reductase (DHAR)	Wheat	Shan et al. 2018
Catalase	Wheat	[62,66]
	Barley	[65]
Guaiacol peroxidase	Wheat	[66]
	Barley	[65]
PAL	Wheat	[66]
GST	Wheat	[66]

**Table 2:** The detected quantitative trait loci (QTLs) for agronomic, physiological and metabolite traits in wheat and barley using bi-parental mapping populations.

Traits	Crop	Chr.	Reference
*Agronomic traits*			
**Grain yield**	Wheat	1B, 1D, 3B, 4A, 6D, 7D	[162]
	Barley	1H, 2H, 3H, 6H	[163,164]
**Grain weight spike^−1^**	Wheat	1B, 1D	[167]
	Barley	2H, 4H, 5H, 6H	[166,171]
**Thousand grain weight**	Wheat	1B, 1D, 2A, 2B, 3A, 3B, 4A, 4D, 6A, 6D, 7B, 7D	[167,172]
	Barley	2H, 5H, 7H	[169]
Grain number m^−2^	Wheat	1B, 5A, 5B, 7D	[162]
**Grain number spike^−1^**	Wheat	1A, 2A, 2B, 3A, 6B	[167,172]
	Barley	2H, 3H, 4H, 5H, 6H	[163,164,166]
**Harvest index**	Wheat	1B, 2D, 4BS, 5A	[167]
	Barley	1H	[169]
Spike number plant^−1^	Wheat	1A, 2A, 2B, 2D, 4B, 5A, 7B	[167]
	Barley	2H, 5H, 6H	[167]
Spikelet compactness	Wheat	1A, 1B, 2B, 5A, 5B, 6A, 6B, 7A	[167,172]
Spikelet number spike^−1^	Wheat	1B, 1D, 2B, 3B, 4B, 5A, 6B, 7D	[167,172]
	Barley	2H, 5H, 6H	[151]
**Sterile spikelet number spike^−1^**	Wheat	7A	[167]
**Fertile spikelet spike^−1^**	Wheat	2A	[167]
**Spike length**	Wheat	2B, 7A, 7B	[167]
**Biomass**	Wheat	1B	[167]
	Barley	2H	[167]
**Shoot biomass**	Wheat	4B	[173]
**Plant height**	Wheat	1B, 4B, 7D	[167,172]
**Spike length**	Wheat	2B, 7A, 7B	[167]
**Lateral spikelet traits**	Barley	1H, 2H, 3H, 5H, 6H	[166]
*Physiological traits*			
**leaf area, growth rate, transpiration efficiency,** **water-use efficiency**	Wheat	2A, 2D, 3A, 4B, 6A,	[169]
**Early vigor, leaf rolling, leaf waxiness,** **leaf chlorophyll content**	Barley	1H, 2H, 3H, 4H, 5H	[155]
**Carbon isotope ratio, osmotic potential,** **chlorophyll content, flag leaf rolling index**	wheat	2B, 4A, 5A, 7B	[174]
**Chlorophyll and chlorophyll fluorescence parameters**	Barley	2H, 4H, 6H, 7H	[175]
**Grain carbon isotope discrimination**	Barley	2H, 3H, 6H, 7H	[176]
**Relative water content**	Barley	6HL	[176]
**Water-soluble carbohydrate**	Barley	4H	[177]
**Water-soluble carbohydrate**	Wheat	1A, 1D, 2D, 4A, 6B, 7B, 7D	[95]
**Stomatal density, index, aperture area,** **length; Guard cell area and length**	wheat	2B, 4AS, 5AS, 7AL, 7BL; 1BL, 4BS, 5BS, 7AS	[178]
**Stomatal conductance,** **Net photosynthetic rate**	wheat	5A, 6B	[167]
**Leaf wilting**	Barley	1H, 2H, 3H, 4H	[179]
**Root length**	Wheat	2D, 4B, 5D, 6B	[173]
	Barley	2H, 3H,5H	[180]
**Root biomass**	Wheat	2D, 4BS	[173]
	Barley	1H, 2H, 3H, 4H, 5H, 7H	[180]
*Metabolite traits*			
**Proline content**	Barley	3H, 4H, 5H, 6H	[179]
**Abscisic acid (ABA)**	Wheat	1B, 2A, 3A, 4D, 5A, 6D, 7B	[181]
**Jasmonic acid (JA), salicylic acid (SA), ethylene**	Wheat	6A	[182]

**Table 3:** The most significant genomic regions with genetic/physical position associated with agronomic, physiological and metabolite traits in wheat and barley using the genome-wide association study (GWAS) approach.

Traits	Crop	Chr. (pos. (cM or bp*))	Reference
*Agronomic traits*			
Grain yield	Wheat	1A(140), 1B(99), 2B(18), 3B(133), 6A(54), 7B(39–40)	[13]
	Barley	1H(133–134), 3H(153–155)	[192]
Grain weight	Wheat	1A(298646355), 1A(522189599), 2A(758448348),2B(47837996), 2D(617414673), 3A(610441472), 4A(7441672), 4A(73454791), 5A(423673926), 6A(615815033), 7A(30902570), 7A(691163940), 7A(14787746)*	[191]
Thousand-grain weight	Wheat	2A(66–70), 3A(69–74)	[13]
	Barley	2H(45–46), 6H(134)	[192]
Grain number spike^−1^	Wheat	2D(128), 4A(132)	[102]
	Barley	3H(126–127), 5H(130–131), 6H(44–45)	[193]
Harvest index	Wheat	3B(194–195), 6B(83)	[185]
	Barley	2H(106–107),	[194]
Spikelet number spike^−1^	Wheat	1B(239), 2B(107), 2D(128), 4B(1), 5B(1), 6B(1)	[102]
	Barley	7H(106–107)	[194]
Biomass	Wheat	1A(85–86), 4B(101), 4D(30), 6B(90)	[185]
	Barley	1H(87–92), 5H(46–47)	[195]
Plant height	Wheat	1A(116–117), 1B(51), 2A(45), 2B(79, 107), 2D(128), 3A(9), 4B(31–32), 5B (65), 6A(12), 7A(88), 7B(59)	[107,108]
	Barley	5H(86–87)	[193]
Spike length	Wheat	1B(184), 2B(107–108), 2D(128), 3A(1), 4B(1), 5B(117), 6A(1), 6B(1), 7A(1), 7D(197~206)	[10,97]
	Barley	1H(64–65), 2H(3–4, 14–15), 6H(95–96)	[193]
*Physiological traits*			
Flag leaf area	Wheat	1A(516732460–575597761), 1B(58989138), 1D(278097355), 2A(29874199), 2A(764065400), 2D(35564010), 4D(54054104), 5A(587423540), 6B(120860110–120860130), 6B(643131336–674558588), 7D(10009696), 7D(558932149), 7D(638535043–638535045)*	[191]
Flag leaf length	Wheat	1B(62791605–667135914), 1D(382219667), 2A(29874199), 2B(140752747), 2D(642055122–71578532), 4A(612662321), 6D(1771825), 6D(463762312), 7B(520419132–68562846)*	[191]
	Barley	2H(117–122), 3H(125–126), 4H(68–69), 6H(95–96)	[193]
Flag leaf width	Wheat	1A(516732460), 1B(453278609–554003233), 1D(16816400), 2B(16009609), 2B(48030550), 2D(32992152), 4B(534722043), 6B(119525401), 6B(220551194), 6B(26200560–320552308), 6B(677338037–73535204), 6D(16376439)*	[191]
	Barley	4H(125), 5H(12)	[193]
Branched root length	Wheat	1A(474451217), 2B(165520954), 6B(292760947, 353776019, 42406493) *	[186]
Root diameter	Wheat	5A(561134164), 5B(699669413– 700035453)*	[186]
Root dry matter	Wheat	1A(508184675), 5B(712600907)*	[186]
Root length	Wheat at flowering	2D(620326979), 3B(757480752), 5B(669373985–669374027), 6A(169248262–169248303), 6D(241296319), 6D(431108774–445773103),7A(94404310)*	[191]
	Barley at seedling	1H(46–48), 2H(12–13, 114)	[8]
Seedling shoot length	Barley	1H(46–48), 2H(12–13, 114)	[8]
Seminal axis length	Wheat	5B(658559755– 711277563)	[186]
Stem water soluble carbohydrates	Wheat	1A(54–58), 1B(159–160), 2B(69–72), 3A(26), 3B(81–83), 3D(130), 4B(62–63)	[190]
Water-soluble carbohydrate accumulation	Wheat	1A)68–69), 1B(11–12), 1D(83–86), 2D (40–41), 4A(62–63)	[9]
Germination and seed viability	Barley	1H(46–48)	[8]
Leaf senescenc	Barley	1H(188–119), 2H(131–132), 3H(142–143), 6H(64–65), 7H(40–41, 81–82)	[196]
Water use efficiency, Water content and Relative water content	Barley	2H(118–119), 3H(24–25), 4H(49–55), 5H(48–49, 147–148)	[197]
Net photosynthesis rate, intercellular CO_2_ concentration, stomatal conductivity	Barley	3H(51–52), 4H(43–49, 51–52)	[197]
Leaf wilting		3H(49–50), 4H(72–73), 5H(53–54), 6H(75–76), 7H(93–94, 125–126)	[198]
Relative water content		2H(51–52, 137–138), 7H(88–89, 125–126, 147–148)	[198]
*Metabolite traits*			
The total content of soluble sugars	Barley	1H(95)	[199]
Osmolality	Barley	1H(116), 2H(51.8), 3H(2.4), 4H(52.3), 5H(46.5), 6H(10.3), 7H(106.5)	[195]
Proline accumulation	Barley	1H(49–50), 2H(137–138), 3H(1–2, 144–145), 7H(147–154)	[198]
